# Blockade of Central Angiotensin II AT_1_ Receptor Protects the Brain from Ischemia/Reperfusion Injury in Normotensive Rats

**Published:** 2014-11

**Authors:** Hamdolah Panahpour, Ali Akbar Nekooeian, Gholam Abbas Dehghani

**Affiliations:** 1 Department of Physiology and Pharmacology, School of Medicine, Ardabil University of Medical Sciences, Ardabil, Iran;; 2 Department of Pharmacology, School of Medicine, Shiraz University of Medical Sciences, Shiraz. Iran;; 3 Department of Physiology, School of Medicine, Shiraz University of Medical Sciences, Shiraz, Iran

**Keywords:** Angiotensin II, Angiotensin AT1 receptor, Candesartan, Stroke, Rat

## Abstract

**Background: **Stroke is the third leading cause of invalidism and death in industrialized countries. There are conflicting reports about the effects of Angiotensin II on ischemia-reperfusion brain injuries and most data have come from chronic hypertensive rats. In this study, hypotensive and non-hypotensive doses of candesartan were used to investigate the effects of angiotensin II AT_1_ receptor blockade by transient focal cerebral ischemia in normotensive rats.

**Methods:** In this experimental study, 48 male Sprague-Dawley rats were randomly divided into four groups (n=12). Sham group, the control ischemic group, and two ischemic groups received candesartan at doses of 0.1 or 0.5 mg/kg at one hour before ischemia. Transient focal cerebral ischemia was induced by 60 minutes occlusion of the middle cerebral artery, followed by 24 h reperfusion. The neurological deficit score was evaluated at the end of the reperfusion period. The total cortical and striatal infarct volumes were determined using triphenyltetrazolium chloride staining technique. Tissue swelling was calculated for the investigation of ischemic brain edema formation.

**Results:** In comparison with the control ischemic group, AT_1_ receptor blockade with both doses of candesartan (0.1 or 0.5 mg/kg) significantly improved neurological deficit and lowered cortical and striatal infarct sizes. In addition, pretreatment with candesartan significantly reduced ischemia induced tissue swelling.

**Conclusion:** Angiotensin II by stimulating AT_1_ receptors, participates in ischemia-reperfusion injuries and edema formation. AT_1_ receptor blockade with candesartan decreased ischemic brain injury and edema and improved neurological outcome.

## Introduction

Ischemic stroke remains one of the main illnesses with a major health and economic impact, and greatly consumes medical resources. With a mortality rate of around 30%, stroke remains the third leading cause of invalidism and death in industrialized countries. Ischemic brain injury is the result of a complex sequence of pathophysiological events that develops over time and space.^1^ Brain edema is a life-threatening complication of cerebral infarction and aggravates the primary ischemic injury to the brain.^2^^,^^3^ It is suggested that the prevention of ischemic brain edema formation reduces neuronal injury following cerebral ischemia.^4^

Beside its multiple physiological functions, renin-angiotensin system (RAS) has been implicated in pathogenesis and outcome of ischemic injuries in vital organs such as heart^5^ and kidney.^6^ Furthermore, it is suggested that RAS may contribute in stroke related pathogenic mechanisms and involve in the ischemic brain damage.^7^ However, the role of RAS in ischemic brain injuries is controversial. Initial studies suggested a protective role for RAS activity in cerebral ischemia but later reports postulated a correlation between Angiotensin (Ang) II and the severity of ischemic injury. Previous studies demonstrated that inhibition of angiotensin converting enzyme by enalapril pretreatment^8^ and treatment.^9^ Furthermore, the reduction of Ang II improved neurological outcome and reduced brain injuries in animal models of focal cerebral ischemia. Most of the physiological action of Ang II has been shown to be mediated by the AT_1 _receptors.^10^ Consequently, this study is designed to investigate the role of RAS activity and its effector peptide, Ang II in conjunction with AT_1_ receptors in brain injuries and edema following transient focal cerebral ischemia in rats.

In this study, candesartan as an AT1 antagonist was used for AT_1_ receptor blocking. It has been demonstrated that candesartan can easily penetrate the blood brain barrier (BBB) to inhibit the effects of central Ang II. The long-lasting blockade of central AT_1_ receptors by candesartan may be attributed to its tight binding and slow dissociation from AT_1_ receptors.^11^^,^^12^ Therefore, the effective and long-lasting blockade of central AT_1_ receptors is produced by candesartan.

The present study uses an intraluminal filament method that induces transient focal cerebral ischemia by middle cerebral artery occlusion (MCAO) in a rat.^8^^,^^9^ This model is less invasive and reperfusion of the ischemic region is feasible. It is comparable with human stroke, in which spontaneous or drug-induced reperfusion occurs. Furthermore, most data about the participation of RAS in cerebral ischemia have originated from chronic hypertensive rats.^13^^-^^15^ Pathologic remodeling of cerebral vessels that occur during chronic hypertension are believed to interfere with the outcomes of neuroprotective agents.^15^ To exclude these possibilities, the model of transient focal cerebral ischemia in normotensive rats was used.

## Materials and Methods

Male Sprague-Dawley rats (300-380g) were obtained from the central animal shelter facility of Shiraz Medical Sciences University (Shiraz, Iran). All protocols of the study were approved by the Institutional Animal Ethics Committee of Shiraz University of Medical Sciences, which follows the National Institutes of Health guidelines for care and use of animals. Animals were housed at room temperature of 22-24°C, humidity of 40-60% and light period of 07.00-19.00 controlled environments. The animals had access to tap water and rat chow, but only had free access to tap water on the night before surgery. They were anesthetized with IP injections of chloral hydrate (400 mg/kg). Two temperature probes were inserted into the left temporalis muscle and rectum to monitor head and core temperatures. Two separate heating lamps were used to keep the core and cranial temperatures at 37±0.5°C.

Experimental Design and Protocol

*Group I *(sham, n=12): rats underwent surgery in the neck region and received a single IV injection of the vehicle (1 ml/kg, 0.1 normal sodium carbonate solution) without being exposed to MCAO.

*Group II *(control ischemic; n=12): rats received a single IV injection of the vehicle 1 h before MCAO. Brain ischemia achieved by 60 minutes MCAO followed by 24 h reperfusion.

*Group III *(candesartan pretreated ischemic rats with 0.1 mg/kg; n=12): rats received a single IV injection of 0.1 mg/kg of candesartan (Toronto Research Chemical Inc, Canada) 1 h prior to MCAO. All procedures, which were performed on rats of group II, were also repeated for this group.

*Group IV *(candesartan pretreated ischemic rats with 0.5 mg/kg; n=12): rats of this group received a single IV injection of 0.5 mg/kg of candesartan 1 h before induction of MCAO. Other procedures were similar to groups II and III.

In some randomly selected rats from each group (n=4), the tail artery was cannulated to record mean arterial blood pressure (MAP) and withdrew 0.3 ml arterial blood for the measurements of physiological parameters including *p*H, *P_O_*_2_, *P*_CO2_, Sa_O2_ and blood glucose. Blood sampling was done at 10 minutes before and 30 minutes after MCAO and 10 minutes after the start of reperfusion. Other animals from each group (n=8) were used for the determination of neurological outcome, infarct size and tissue swelling.

Induction of Transient Focal Cerebral Ischemia

Transient focal cerebral ischemia induced by occluding the middle cerebral artery (MCA) using the intraluminal filament method described previously.^16^ In brief, a midline incision was performed in the ventral surface of the neck. Afterwards, the right common carotid artery (CCA) was freed from surrounding fascia and vagus nerve dissected to reach the bifurcation of external and internal carotid arteries. The occipital artery and superior thyroid branches of the external carotid artery (ECA) were isolated and closed. Subsequently, ECA was ligated permanently and the internal carotid artery (ICA) dissected free to the level of pterygopalatine artery. Afterwards, a poly-L-Lysine coated nylon 3-0 monofilament thread was inserted, via proximal ECA, into ICA and gently moved forward to the circle of Willis to reach and occlude MCA. After 60 minutes ischemia, reperfusion was established by pulling out the nylon thread. After reperfusion phase, the vessels were then tied up and the incisions were sutured. The animals were allowed to recover from anesthesia, and were then kept in separate cages. Twenty-four hours after termination of MCAO, neurological activity was evaluated and animals were then sacrificed under deep anesthesia by a bolus of injection of sodium thiopental solution. The brain was removed and prepared for the determination of the infarct volumes.

Measurement of Infarct Size and Tissue Swelling

The brain was gently removed and placed in ice-cold saline for 5 minutes to solidify for coronal sectioning using a brain matrix. The brain was sectioned into six 2-mm-thick slices. The slices were stained by immersing in 10 ml 2% Triphenyltetrazolium chloride (TTC) solution, which had been placed in a water bath of 37°C for 30 minutes. The stained slices were kept in 10% buffered formalin for 24 hours. Afterwards, the slices were photographed with a digital camera connected to a PC computer. Infarct area of each slice was measured in mm^2^ using an Image Analyzer Software (NIH Image Analyzer). The infarct volume in mm^3^ of each slice was calculated by multiplying the obtained infarct areas by the slice thickness. Finally, the infarct volumes of six slices were summed up to calculate the total infarct size of each brain.^17^ Since brain edema could significantly affect the accuracy of the estimation of the infarct volumes, the calculated infarct volumes were then corrected for brain edema according to equation 1. The tissue swelling induced in the lesioned hemispheres of the TTC staining method was evaluated as in equation 2.^15^

(1) Corrected infarction volume=[NHV–(LHV–MLV)]

(2) %Tissue swelling=[(LHV–NHV)/NHV] ×100 

NHV: Non-lesioned hemisphere volume (left hemisphere)

LHV: Lesioned hemisphere volume (right hemisphere)

MLV: Measured lesion volume

Statistical Analysis

All statistical analyses were performed by SPSS software (SPSS for Windows version 20) Most of the data are presented as mean±SEM and the significance of differences was evaluated using one-way analysis of variance (ANOVA) followed by Tukey’s test. Neurological deficit values are given as median values with quartile range (25-75%). Significant differences were analyzed by Kruskal–Wallis one-way ANOVA test. Statistical significance was accepted at P<0.05. 

## Results

Physiological Parameters

The physiological parameters measured in this study such as P_aO2_, S_a_O_2_, P_a_CO_2_, pH, blood glucose, head and core temperatures are summarized in table 1. There was no significant difference between these parameters using intra-groups or inter-groups comparison.

**Table 1:** Physiological parameters in the studied groups

**Parameters Name**	**Pre ischemia**			**During Occlusion**			**During Reperfusion**		
	**group II**	**group III**	**group IV**	**group II**	**group III**	**group IV**	**group II**	**group III**	**group IV**
PaO_2(mmHg)_	116± 8	105±21	127±11	103±11	105±5	110±15	114±9	114±11	104±12
SaO_2(%)_	98±0.4	96.5±1.3	98.3±0.4	96.7±1	97.3±0.3	96.6±1.8	97.8±0.5	97.9±0.6	96.3±1.8
PaCO_2(mmHg)_	42.5±0.7	40±2.8	42.1±1.4	40.2±0.7	39.9±2.2	39±2	41.1±1.6	41.1±2	41.2±1.6
pH	7.32±0.01	7.33±0.01	7.31±0.01	7.3±0.01	7.31±0.01	7.3±0.02	7.3±0.01	7.32±0.01	7.29±0.01
Blood Glucose (mg/dl)	137±15	141±10	124±17	129±10	135±9	121±16	133±8	142±14	122±18
Body Temperature	37.1±0.06	37.3±0.15	37.3±0.14	37.4±0.1	37.4±0.09	37±0.05	37.2±0.1	37.5±0.09	37.4±0.09
Head Temperature	37.1±0.05	37.4±0.1	37.4±0.13	37.5±0.09	37.4±0.08	37.5±0.1	37.4±0.1	37.5±0.05	37.5±0.09
MAP (mmHg)	91.9±3	78.3±5	57±11*	92.3±2	83.5±5	69±3	92±3	74.5±8	68.4± 9

Values are mean±SEM of four rats in each group; *Significant compared with the respective control rats (group II), P<0.05. MAP: mean arterial blood pressure

Mean Arterial Blood Pressure

There was no significant difference among the mean arterial blood pressure (MAP) of ischemia reperfused (IR) rats received vehicle (group II) or candesartan at 0.1 mg/kg (group III) at 10 minutes before MCAO, 30 minutes after MCAO or 10 minutes after the onset of reperfusion. Whereas the MAP of IR rats received candesartan at 0.5 mg/kg (group IV) were significantly lower than those of IR rats received vehicle only at 10 minutes before MCAO (P=0.03, table 1).

Neurological Deficit Score (NDS) 

The NDS of IR rats received vehicle were significantly higher than that of sham-operated rats. However, there were significant improvements in NDS of ischemic rats treated with 0.1 mg/kg or 0.5 mg/kg of candesartan. The median (25–75% quartile range) of the total deficit score for control ischemic rats (group II) was 3 (2–3) compared with 1 (1.0–2) in group III (P=0.008) and 1 (1.0–2) in group IV (P=0.003) that received candesartan at 0.1 mg/kg or 0.5 mg/kg respectively (Kruskal–Wallis one-way ANOVA test).

Cerebral Infarct size 

**Figure 1:**
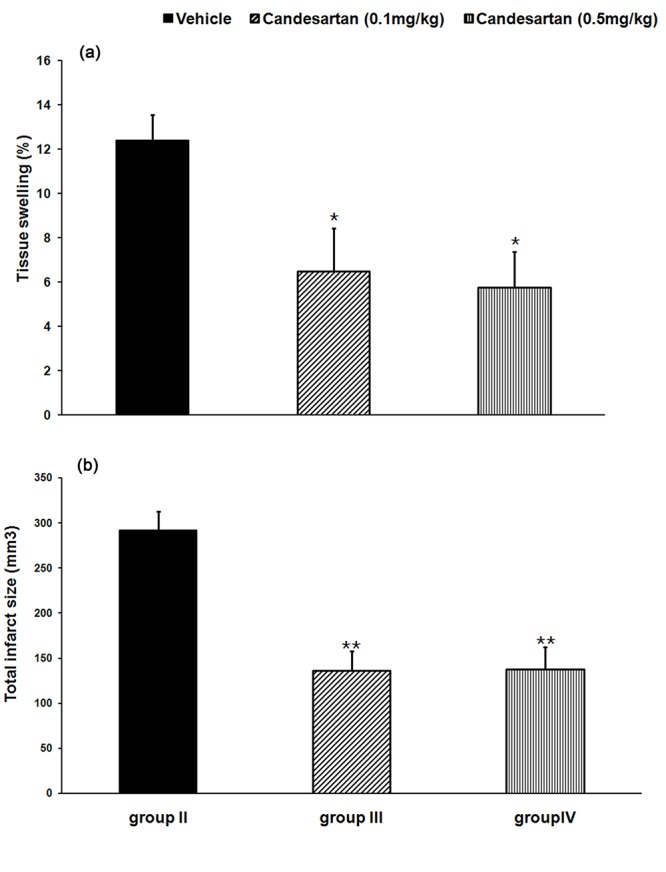
Tissue swelling (a) and total infarct size (b) in the studied groups. Values are mean±SEM of eight rats in each group. *P<0.05, **P<0.001 significant as compared with the respective vehicle-treated control rats (group II).

**Figure 2:**
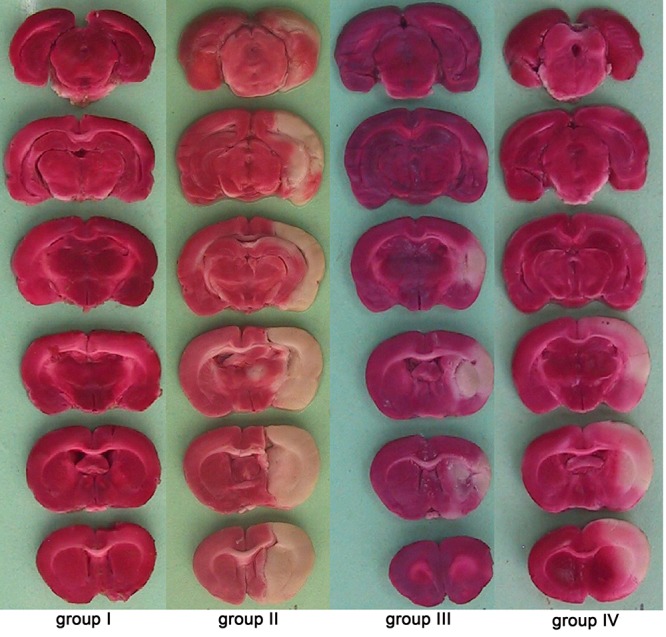
Representative brain slices stained with TTC in the studied groups. Ischemic regions are white and non-ischemic regions are red colored.

**Figure 3:**
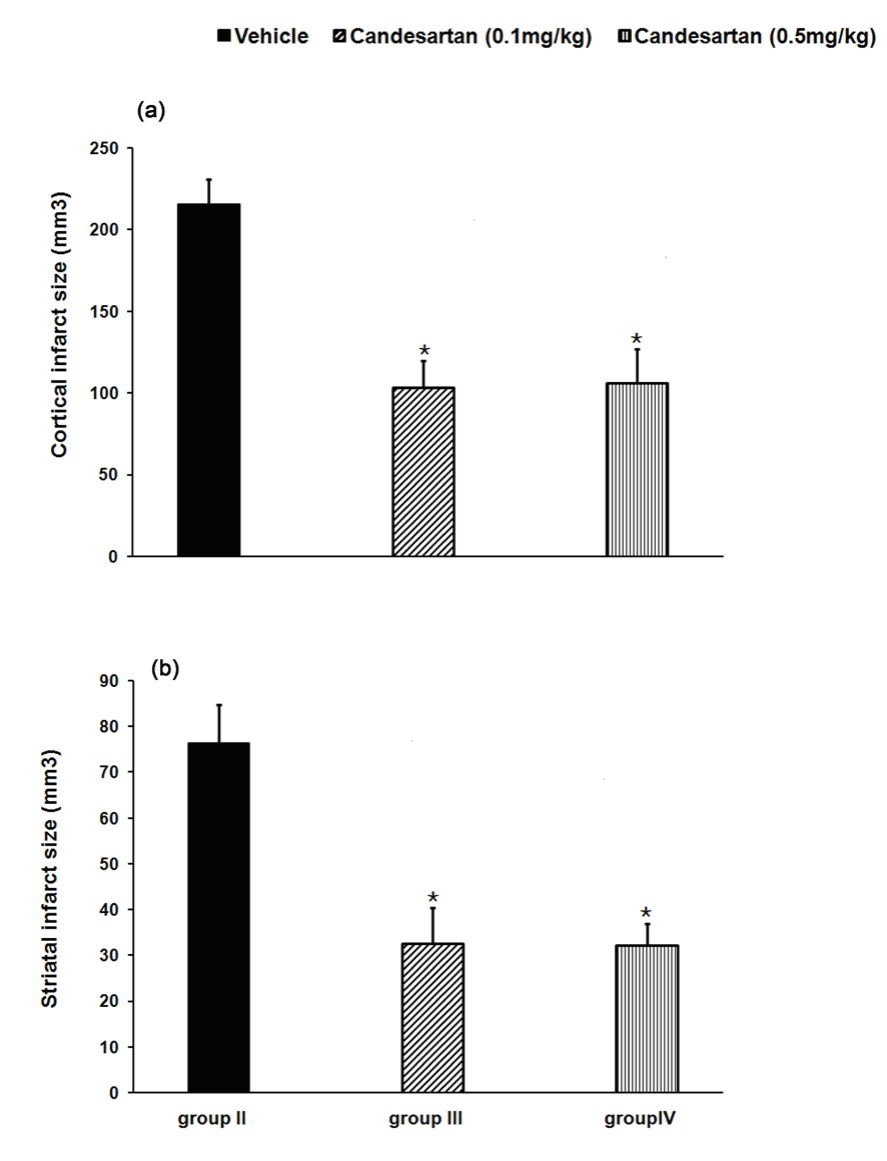
Cortical (a) and striatal (b) infarct sizes in the studied groups. Values are mean±SEM of eight rats in each group. *P<0.01 significant as compared with vehicle-treated control rats (group II).

The sham-operated rats had no cerebral infarction. In comparison with cerebral total infarct size of IR rats received vehicle (group II: 292±21 mm^3^), IR rats received candesartan at 0.1 or 0.5 mg/kg had significantly lower total infarct size (group III: 136±21 mm^3^ P=0.001, and group IV: 138±24 mm^3 ^respectively, P=0.001, figure 1). Furthermore, pre-ischemic AT1 receptor blockade with candesartan at 0.1 or 0.5 mg/kg, significantly reduced cortical and striatal infarction sizes compared to IR rats received vehicle (P=0.001 for both figure 2 and figure 3).

Tissue Swelling

Occurring ischemia produced considerable tissue swelling in IR rats received vehicle (group II: 12.4±1.1%). Pre-ischemic AT1 receptor blockade with candesartan significantly decreased tissue swelling by 47.6 % at 0.1 mg/kg (group III, P=0.04) and 53.6% at 0.5 mg/kg (group IV, P=0.02) respectively (figure 1).

## Discussion

The results of this study revealed that the blockade of AT_1_ receptors, reduced cortical and striatal infarct sizes and improved neurological motor deficits. These findings are in agreement with reports from other investigators demonstrating that AT_1_ receptor blockade with candesartan reduced infarct size in transient cerebral ischemia in normotensive rats,^18^ or permanent focal ischemia in adult spontaneously hypertensive rats.^15^ Transient or permanent MCA occlusion in AT_1_ knockout mice, as compared with normal type, produced smaller core in the ischemic area with a much larger penumbra.^19^ In contrast, Groth et al.^20^ and Sugawara et al.^21^ reported that inhibition of AT_1_ receptors by pretreatment of the animal with candesartan did not reduce ischemic brain injuries in the rat. This controversy is believed to be related to hypotension observed before, during and after ischemia.^20^ The present study also showed that the protective effects of candesartan against brain injuries and edema at high dose (0.5 mg/kg) was not significantly different with non-hypotensive dose of this drug (0.1 mg/kg). Candesartan at 0.5 mg/kg could significantly decrease MAP before ischemia. During cerebral ischemia, the mechanisms regulating CBF are impaired leaving the dependency of local CBF on arterial blood pressure.^22^ Hence severe hypotension, possibly did mask the protective effects of AT_1_ receptor blockade. It seems that there is a positive correlation between the level of MAP and the protective effects of AT_1_ receptor blockade and profound drop in MAP hypoperfuses the ischemic area of the brain and prevent the protective effects of AT_1_ receptor blockade against IR injuries.

Various mechanisms might be responsible for the beneficial effects of AT_1_ receptor blockade in brain ischemia. Such effects might be partly attributed to their stabilization actions on the impaired cerebrovascular autoregulation in penumbra. In addition, they might be due to effects on the nucleus solitary tract that contributes to the regulation of CBF by a reduction of central sympathetic tone.^23^ Anti-apoptotic mechanisms might be involved in the protective effects of AT_1_ receptor blockade. Ang II, via activation of AT_1_ receptors, may provoke apoptosis of ischemic neurons.^24^ Thus, anti- apoptotic effects of AT_1 _receptor blockade may have beneficial effects on ischemic brain injury.

The beneficial effects of AT_1_ blockade might also be attributed to the reduction of reactive oxygen species (ROS) production.^21^ Cerebral ischemia is associated with excessive production of ROS, especially superoxide. The production of ROS initiates chain reactions causing cellular macromolecular damage, and promotes the mitochondrial apoptosis pathway, which ultimately leads to cell death.^25^The stimulation of AT_2_ receptors might also be involved in the beneficial effects of AT_1_ receptor blockade. The blockade of AT_1_ receptors may slow down the neurodegenerative events in ischemic brain tissues by allowing Ang II to increase the stimulation of AT_2_ receptors.^26^

Brain edema is a life–threatening complication of cerebral infarction and aggravates the primary ischemic injury to the brain by negatively affecting the perfusion of penumbra due to the compression of cerebral vasculature via increased intracranial pressure and herniation.^2^^,^^3^ The results of the present study demonstrated that induction of focal cerebral ischemia significantly increased tissue swelling of ischemic hemisphere. While, pre-ischemic blocking of AT_1_ receptors by candesartan significantly reduced swelling in the ischemic hemisphere and decreased ischemic edema. Other reports about the evaluation of tissue swelling of the ischemic hemispheres, from TTC method^15^^,^^23^^,^^27^ supports the results of the present study. The protective effects of AT_1_ receptor blockade on ischemic brain edema were demonstrated in other experimental models and animal species. Blezer and colleagues showed that AT_1_ receptor inhibition by non-hypotensive dose of losartan reduced cerebral edema and markedly prolonged survival in SHR.^28^ In addition, the inhibition of AT_1_ receptors by losartan prevented brain edema following global cerebral ischemia in the cat.^29^ These results support the conclusions of the present study that Ang II and AT_1_ receptors might be involved in the formation of brain edema during I/R injuries.

The mechanisms of the beneficial effects of AT_1_ receptor blockade on ischemic brain edema have not been fully elucidated. It seems that non-hypotensive mechanisms play a major role, since the present study as well as others^28^^,^^29^ showed that the protective effects of AT_1_ receptor blockade was achieved without hypotension. Mechanisms such as normalizing of endothelial NO synthase activity, and reduced cerebrovascular inflammation might also be involved in such protective effects.^30^ Furthermore, Ang II via AT_1_ receptors may enhance cerebrovascular permeability and edema by production of ROS and matrix metalloproteinases.^31^^,^^32^ Thus, blocking of AT_1_ receptors, may reduce ischemic edema by the protective effects on BBB integrity and lowering its permeability during ischemia. However, the clarification of this postulation needs more study.

## Conclusion

The results of this study demonstrate that RAS participates in the exacerbation of cerebral I/R injuries, and Ang II may play an important role as mediator in ischemic brain injury by stimulating of AT_1_ receptors. Hence, the inhibition of RAS by an AT_1_ receptor blocker reduced cerebral infarction size, tissue swelling and improved neurological motor activity in rats exposed to transient MCA occlusion.
